# A spontaneous suprachoroidal haemorrhage: a case report

**DOI:** 10.1186/1757-1626-2-185

**Published:** 2009-11-06

**Authors:** Aman Chandra, Allon Barsam, Charles Hugkulstone

**Affiliations:** 1 Vitreoretinal department, Moorfields Eye Hospital, City Road, London, EC1V 2PD, UK; 2 Department of Ophthalmology, Queen Mary's Hospital, Sidcup, Kent DA14 6LT, UK

## Abstract

**Introduction:**

We present a case of spontaneous suprachoroidal haemorrhage in a patient taking Warfarin. This is only the second case reported of a patient whose anticoagulation was within the therapeutic range.

**Case presentation:**

An 84 year old white male with a history of end stage atrophic age related macular degeneration presented with angle closure glaucoma. The patient was taking warfarin and had a therapeutic International Normalized Ratio (INR). Ultrasound examination revealed a spontaneous suprachoroidal haemorrhage.

**Conclusion:**

Anticoagulation is common in those with cardiovascular disease, which increases the risk of haemorrhagic complications. These patients are also more likely to suffer from age related macular degeneration. Suprachoroidal haemorrhage should be considered in such patients presenting with suspicious signs and a low threshold should be had for investigating for this condition in such circumstances. Early detection may reduce the morbidity.

## Introduction

Suprachoroidal haemorrhages are rare, and most commonly associated with intraocular surgery. Anticoagulant therapy and cardiovascular disease are known risk factors. Spontaneous suprachoroidal haemorrhages (SSCH) have only been reported a few times and have poor prognoses. We report of such a case, and in particular only the second associated with anticoagulation within the therapeutic range.

## Case presentation

**Figure 1:**
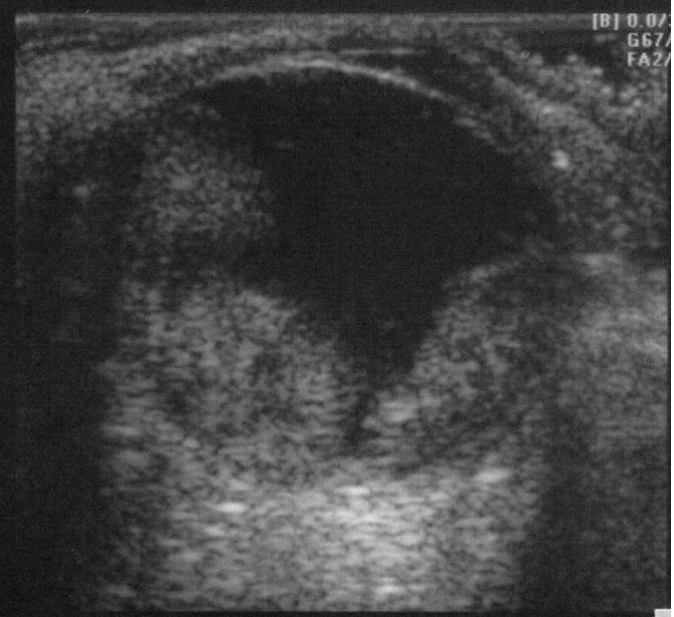
**B-scan ultrasonography of the left eye at presentation**.

An 84 year old man with a history of bilateral end stage atrophic ARMD presented with a gradual loss of peripheral vision in his left eye over a few days, associated with worsening pain in and around that eye. His medical history included treated hypertension, cardiac bypass surgery and a previous pulmonary embolus for which he was taking Warfarin. On examination, his vision was counting fingers (CF) at 3 metres in the right eye, and no perception of light (NPL) in the left. He had a markedly injected left eye with corneal oedema, a shallow anterior chamber and a completely closed drainage angle. The anterior chamber of the right eye was deep with an open angle. The intraocular pressures (IOP) were 14 mmHg on the right and 44 mmHg on the left. An ultrasound scan of the left eye demonstrated a suprachoroidal haemorrhage (Figure 1). His blood pressure was 189/90. A full blood count and coagulation tests revealed low platelets of 110 × 10^9^/L (normal range 150-400) and an International Normalization Ratio (INR) of 4.0 (therapeutic range 3.0-4.0). He was admitted and given 500 mg Acetazolamide intravenously and commenced on G. Timolol 0.5% twice daily, G. Latanoprost at night, G. Atropine 1% three times daily and G. Dexamethasone four times daily to the left eye, whilst his Warfarin was continued. His intraocular pressure remained stable at 35 mmHg over 2 days, and he was discharged on the same topical treatment, plus oral Acetazolamide 250 mg four times daily, which was stopped after two weeks. At follow up one month later, his left visual acuity was unchanged at NPL, the IOP in this eye was 8 mmHg on topical treatment only and he remained pain free.

## Discussions

Suprachoroidal haemorrhage (SCH) is a rare condition, usually associated with intraocular surgery. Ophthalmic risk factors include glaucoma, aphakia, elevated IOP, axial myopia and inflammation [1]. Advanced age, hypertension and atherosclerosis are recognised systemic risk factors [1]. It is thought that ocular hypotony, either during surgery or otherwise, is the major precipitating factor in SCH [2]. Our patient presented with angle closure secondary to spontaneous suprachoroidal haemorrhage (SSCH). There are previous reports of SSCH in patients after valsalva manoeuvre [3], with ocular hypertension and open angles and high myopia [4] without angle closure glaucoma. In those cases associated with thrombolytic therapy [2,5,6], the two after myocardial infarction [2,5] presented with acute angle closure whilst the report after a cerebrovascular accident had a narrow angle with normal IOP [6].

Fragile choroidal and posterior ciliary vasculature may have an aetiological role to patients with high myopia [4] aphakia, intraocular hypertension, inflammation, systemic hypertension and arteriosclerosis [1]. It has been suggested that choroidal vascular abnormalities secondary to ARMD may also predispose to spontaneous haemorrhage [7]. Although not assessed, we hypothesise that our patient may thus have had underlying choroidal abnormalities, secondary to ARMD, but he had no other ocular risk factors. He did have significant systemic risk factors for SSCH including advanced age, hypertension and anticoagulant therapy. Although our patient had a mild thrombocytopenia, it is unlikely to have had a significant role in the aetiology of the SSCH, as bleeding is uncommon with platelet counts above 50 × 10^9^/L, whilst severe spontaneous bleeding is unusual with platelet counts above 20 × 10^9^/L [8]. Our patient's INR was within the therapeutic range and to the best of our knowledge, this is only the second case of SSCH reported with a therapeutic INR. Alexandrakis *et al. *[7] reported a patient with exudative ARMD developing SSCH whilst being treated with Warfarin with an INR within the therapeutic range. Knox *et al. *[9] presented a similar case but whose INR was more than double the therapeutic range. They suggest that the disciform bled, with subsequent extension due to the increased clotting time.

Angle closure may occur in SSCH due to forward displacement of the lens- iris diaphragm. This can lead to a diagnostic dilemma, as the presentation may mimic pupil block angle closure glaucoma and distinguishing the two conditions is very relevant. Pilocarpine is contraindicated in spontaneous SCH angle closure because it moves the lens-iris diaphragm forward. Medical management involves topical betablockage and oral carbonic anhydrase inhibitors [3]. Surgical intervention is usually deferred for 2 weeks, although the timing is controversial [2]. Chorich et al [10] report a case in which urgent surgical drainage of a suprachoroidal haemorrhage resulted in vision improvement from no perception of light to perception of light. Although this is only a very modest improvement, it may suggest that early detection and intervention may reduce morbidity in these cases. Due to our patient's poor visual potential, only medical management was undertaken.

## Conclusion

Anticoagulation is common in the elderly, particularly amongst those with cardiovascular disease, which increases the risk of haemorrhagic complications. These patients are also more likely to suffer from age related macular degeneration. The physician therefore needs to have a high level of suspicion in elderly patients on anti-coagulation presenting with suspicious signs. This should be particularly the case in those presenting with open angles on the contralateral side, and the physician should have a low threshold for performing B-scan ultrasound in such cases. Early detection may reduce the morbidity of this condition.

## Abbreviations

INR: International Normalization Ratio; SCH: Suprachroidal Haemorrhage; SSCH: Spontaneous Suprachoroidal Haemorrhage; RE: Right Eye; LE: Left Eye; VA: Visual Acuity; CF: Counting Fingers; HM: Hand Movements; IOP: Intraocular Pressure; ARMD: Age Related Macular Degeneration.

## Competing interests

The authors declare that they have no competing interests.

## Authors' contributions

AC wrote the manuscript and contributed to the management of the patient. AB helped write the manuscript and management of the patient. CH helped write and reviewed the manuscript. All authors have read and approved the final manuscript.

## Consent

Written informed consent was obtained from the patient for publication of this case report and accompanying images. A copy of the written consent is available for review by the Editor-in-Chief of this journal.
